# Copper Oxide Nanoparticles Impact Several Toxicological Endpoints and Cause Neurodegeneration in *Caenorhabditis elegans*

**DOI:** 10.1371/journal.pone.0167613

**Published:** 2016-12-02

**Authors:** Michael J. Mashock, Tyler Zanon, Anthony D. Kappell, Lisa N. Petrella, Erik C. Andersen, Krassimira R. Hristova

**Affiliations:** 1 Department of Biological Sciences, Marquette University, Milwaukee, Wisconsin, United States of America; 2 Department of Molecular Biosciences, Northwestern University, Evanston, Illinois, United States of America; University of Louisville School of Medicine, UNITED STATES

## Abstract

Engineered nanoparticles are becoming increasingly incorporated into technology and consumer products. In 2014, over 300 tons of copper oxide nanoparticles were manufactured in the United States. The increased production of nanoparticles raises concerns regarding the potential introduction into the environment or human exposure. Copper oxide nanoparticles commonly release copper ions into solutions, which contribute to their toxicity. We quantified the inhibitory effects of both copper oxide nanoparticles and copper sulfate on *C*. *elegans* toxicological endpoints to elucidate their biological effects. Several toxicological endpoints were analyzed in *C*. *elegans*, including nematode reproduction, feeding behavior, and average body length. We examined three wild *C*. *elegans* isolates together with the Bristol N2 laboratory strain to explore the influence of different genotypic backgrounds on the physiological response to copper challenge. All strains exhibited greater sensitivity to copper oxide nanoparticles compared to copper sulfate, as indicated by reduction of average body length and feeding behavior. Reproduction was significantly reduced only at the highest copper dose, though still more pronounced with copper oxide nanoparticles compared to copper sulfate treatment. Furthermore, we investigated the effects of copper oxide nanoparticles and copper sulfate on neurons, cells with known vulnerability to heavy metal toxicity. Degeneration of dopaminergic neurons was observed in up to 10% of the population after copper oxide nanoparticle exposure. Additionally, mutants in the divalent-metal transporters, *smf-1* or *smf-*2, showed increased tolerance to copper exposure, implicating both transporters in copper-induced neurodegeneration. These results highlight the complex nature of CuO nanoparticle toxicity, in which a nanoparticle-specific effect was observed in some traits (average body length, feeding behavior) and a copper ion specific effect was observed for other traits (neurodegeneration, response to stress).

## Introduction

Nanoparticles (NPs), microscopic particles, with at least one dimension less than 100 nm, are increasingly incorporated into commercial products. NPs have high surface area to size ratios, conferring unique material properties compared to micron particles with the same chemical composition. Copper oxide (CuO) NPs have strong superconductive properties so they are included in many different consumer electronics including gas sensors, batteries, and solar cells [1]. CuO NPs are also incorporated into several personal care and household products because of their bactericidal properties. Introduction into the environment and unintended human exposure will increase in occurrence as nanoparticle manufacturing output increases. Estimations by Keller *et al*. [2] suggest between 260,000–309,000 metric tons of nanoparticles, of which ~200 metric tons contain Cu and CuO, were produced in 2010 and between 63–91% may have eventually ended up in landfills [3]. The risk of environmental contamination is especially important for copper materials as Cu ions and CuO nanoparticles display toxicological responses towards aquatic species [4] and earthworms [5]. Organic acids found in natural environments affect CuO NP mobility and aggregation behavior via ligand-promoted dissolution [6]. Additionally, the adsorption of organic acids to NPs surface results in dissolution and the release of metal ions [4].

CuO NPs release Cu ions into natural and synthetic aqueous media, meaning the observed cytotoxic or inhibitory effects could be the result of either the nanoparticle structure or the free Cu ions [7,8]. Toxicity caused by CuO nanoparticles and free Cu ions are commonly related to the high redox activity of Cu [4,9]. Free Cu ions can produce hydroxyl radicals at the exterior of cells causing damage to cell membranes [4,9]. Excess levels of heavy metals can also lead to disruption of protein structure from competition and displacement of cations bound within proteins [10]. The released copper ions from CuO NPs has been suggested as the sole source of toxicity in earthworms, several algae and crustacean species, *Escherichia coli*, and human cell lines [1]. By contrast, other studies suggest the NP component also contributes to inhibition of cellular metabolic activity [8] and cytotoxicity in yeast [11] and plants [12]. Toxicological endpoints of CuO NPs have been reported for many model organisms, including bacteria [7], yeast [11], *Oligochaeta* [13], and human cell lines [14]. *Caenorhabditis elegans* was chosen as a useful environmental model to further define CuO NP toxicity because nematodes are important for decomposition of organic matter within soil of natural ecosystems.

*C*. *elegans* are a well-established nematode model organism used in many studies because of low culture cost, short lifespan, and the conservation of many genes and processes with humans. Although the toxic effects of CuO NPs on nematodes has not been extensively studied [15], copper ions from copper salts have been shown to be toxic to several species, including *C*. *elegans* [16], *Panagrellus redivivus*, and *Pristionchus pacificus* [17]. Exposure of laboratory-adapted *C*. *elegans* to toxic levels of copper sulfate was observed to reduce brood size and life span while slowing development [18–20].

Numerous studies in the last decade have observed that the N2 Bristol strain has become genetically modified after many years of culturing under laboratory conditions [21–23]. Several newly derived alleles have appeared in the N2 strain, developed over many years of continuous laboratory culture, resulting in altered physiology and behavior compared to wild nematode strains. These laboratory-adapted alleles could subsequently affect interpretations of toxicity testing [24]. Using the N2 strain, we can compare this strain with more recently isolated *C*. *elegans* strains that may lack these laboratory-adapted genetic changes. Similarly, a recent study using three laboratory cultures and seven wild populations of the amphipod *Hyalella azteca* showed a more than 550-fold variation in sensitivity to pyrethroid insecticides [25] among these diverse strains. When addressing the effects of copper exposure on reproduction and lifespan traits within five populations of brine shrimp, *Artemia*, the environmental component was found to be the major factor explaining the variance in the traits [26] indicating the importance at measuring toxicity in populations from different environments and multiple traits. Evidence of clonal variation in sensitivity to the fungicide azoxystrobin was found in a study examining several clones of *Daphnia magna* isolated from different lakes [27]. These studies highlight the importance of interspecies and intraspecies variation in toxicological studies.

Genomic variation has been observed amongst different wild *C*. *elegans* isolates from a genome-wide assessment of 202 strains revealing 97 distinct genome-wide haplotypes [28]. A recent study of 208 wild *C*. *elegans* strain whole genomes suggested genetic variation in genes that resulted in the lengthening of telomeres did not significantly affect nematode longevity [29]. Recent studies have revealed unexpected variation in both fertility and oocyte function in wild strains of *C*. *elegans* upon exposure to high temperature [30], in responses to the anthelmintic avermectin [31], and in mitotic spindle structure and function [32]. The effects of environmental and chemical stressors, including CuO NPs, to *C*. *elegans* wild strains in comparison to a laboratory-adapted strain is important to consider [23].

*C*. *elegans* is an excellent model to study neuronal health as it offers a relatively simple nervous system [16]. This use has been exploited to study neurodegeneration induced by metals [16,20,33]. Neurodegeneration is a sensitive toxicological endpoint of copper ion exposure [34]. However, the effect of CuO NPs on neurodegeneration in *C*. *elegans* has not been investigated. It is critical for cells to maintain strict regulation and control of copper homeostasis for normal neurological development [35]. Heavy metals, including copper, have been shown to affect neurons by depleting cellular energy through decreased mitochondrial function, increased oxidative stress, or activation of the necrosis or apoptosis pathways [16]. *C*. *elegans* has dopaminergic neurons that allow investigation of the effect of CuO NPs on this class of neurons. A known consequence of Parkinson’s disease is the death of dopaminergic neurons [16]. Damage or loss of these neurons in *C*. *elegans* can cause changes in behavior and possibly alter their responses to environmental stimuli [16].

The goal of the present study was to evaluate CuO NPs inhibitory effects on the model organism *C*. *elegans*. In order to explore the influence of genetic background regarding the response to copper challenge, we examined three wild *C*. *elegans* isolates together with the Bristol N2 laboratory strain. We hypothesized that the established dissimilarities in the genotypes of these wild strains will result in differential sensitivity to copper when compared to the laboratory-adapted N2 strain. Several toxicological endpoints were analyzed using a high-throughput phenotypic screening process developed using the COPAS BISORT large particle nematode sorter [30] to quantify the effects of CuO NPs in comparison to soluble copper salt. Physiological effects of CuO NPs on *C*. *elegans* were investigated through the use of strains with dopaminergic neurons expressing GFP to visualize neurodegeneration after copper exposure. Two knockout mutants of the divalent-metal ion transporters, *smf-1* or *smf-2*, were used to determine if the toxic effect of CuO NPs observed in *C*. *elegans* is SMF transporter dependent. To examine the potential effect of copper as a stressor, a reporter strain with GFP expression driven by an *hsp-16*.*2* stress inducible promoter was examined after CuO NPs and copper sulfate exposure. This work represents one of the first to address and quantify CuO NPs effects in *C*. *elegans*.

## Materials and Methods

### *Caenorhabditis elegans* strains and cultivation conditions

All *C*. *elegans* strains were cultured on nematode growth medium (NGM) plates seeded with the *Escherichia coli* strain OP50. *C*. *elegans* strains were transferred twice a week and stored at 20°C according to the standard method previously described by Brenner [36]. Three wild strains and N2 mutants were also assayed in addition to the N2 wild-type strain. The N2 strain was a kind gift of Dr. R. Stuart (Marquette University, Milwaukee, WI, USA). The wild strains CB4856, DL238, and JU258 were kind gifts of Dr. E. Andersen (Northwestern University, Evanston, IL, USA) and their genetic characteristics were described previously [28]. Strains CB4856 and DL238 are among the most highly diverged wild strains in the species [28,37,38]. The wild JU258 strain is more related to the N2 strain than the other two wild strains but nonetheless divergent [28]. The transgenic strains RJ907 (P_*dat-1*_::GFP; *smf-1(eh5)*) and RJ938 (P_*dat-1*_::GFP*; smf-2(gk133)*), each containing GFP expression controlled by the *dat-1* promoter, were kind gifts of Dr. R. Nass (Indiana University School of Medicine, Indianapolis, Indiana, USA). The BY250 strain (P_*dat-1*_::GFP; N2 wild-type) was a kind gift of Dr. R. Blakely (Vanderbilt University, Nashville, TN, USA). Specifics on the construction of these transgenic *C*. *elegans* lines can be found in Nass *et al*. [39]. A reporter strain KC136: *wxIs39* (*pRF4*+ *pPD99*.44), with GFP expression controlled by the heat-shock protein (HSP) *hsp-16*.*2* promoter, was a generous gift of Dr. K. L. Chow (Hong Kong University of Science and Technology, Clear Water Bay, Kowloon, Hong Kong) [40]. Nematodes were exposed to copper sulfate or CuO NPs for 96 hours in K medium [17] with HB101 bacterial lysate suspended within the medium to prevent nutrient deprivation [23].

### Nanoparticle characterization

The uncoated copper oxide (CuO) nanoparticles (NPs) in this study were purchased from Melorium Technologies. Transmission electron microscopy was performed on the CuO NPs, and they were found to be uniform in both shape and size, spherical with an average diameter of 28 nm [8]. The CuO NPs were diluted into sterile K medium at optimal concentrations (2.5 mg CuO/L) for hydrodynamic characterization using NS500 platform (Nanosight Ltd). NPs solutions were injected into the viewing chamber equipped with 640-nm laser and measurements were taken at room temperature. The Nanosight measurement was performed as previously without modification [8]. To determine the CuO NPs average diameter, the nanoparticle tracking analysis 2.0 Build 127 analytical software was used. Nanoparticles smaller than 10 nm are not detected because of the minimum detection limit of the Nanosight instrument.

### NPs dispersion

Stock solutions of CuO NPs were stored in sterile deionized water at 8,000 mg/L wrapped in aluminum foil to prevent exposure to light. Stock solutions were dispersed using a probe sonicator (Branson Digital Sonifer, 450W) for five minutes prior to dilution to working concentrations for exposure. The concentration of total Cu ions within the CuO NPs and copper sulfate stock solutions were determined to be 7,912 and 7,826 mg Cu/L respectively, using inductively coupled plasma-mass spectrometry (ICP-MS, 7700x ICP-MS with autosampler, Agilent Technologies).

### NPs dissolution

To define the concentration of copper ions (Cu^2+^) released from CuO NPs suspended in K medium, aliquots of the exposure solution were collected at various time points (immediately after dispersion, 4 hours, 24 hours, 48 hours, and 96 hours after dispersion). NP aliquots were centrifuged at 14,000 rpm for 30 minutes and the supernatant was subsequently filtered with a low protein binding syringe filter (0.1 μm Supor Acrodisc PALL Life Sciences) to remove nanoparticle aggregates and agglomerates prior to storage at -20°C. Experiments addressing Cu ion adsorption suggest a fraction of ions are lost after the filtration step but not after the centrifugation step (Fig A in S2 File). The total Cu ions released from CuO NPs in K medium and in the stock copper solutions were determined using ICP-MS. In order to evaluate the contribution of the copper ions in the observed CuO nanoparticle inhibitory effect, an equal molar copper sulfate solution was used as a proxy for dissolute copper in an exposure scenario.

After centrifugation and filtering of the supernatant, the solutions were digested with an equal volume of 70% (w/v) HNO_3_. Digested samples were incubated for 2 hours at 65°C and stored at 4°C in acid-washed glass vials. Prior to analysis, the samples were diluted to 2% HNO_3_ with 0.5% HCl. ICP-MS detects total copper, regardless of ionic species, and will quantify Cu ions bound to salts or organic material and remaining CuO NPs within the supernatant.

### High-throughput endpoint assays for nematodes

All *C*. *elegans* strains were exposed to copper in the form of CuO NPs or copper sulfate at 3.8, 7.9, and 15.9 mg Cu/L for 96 hours. CuO NPs at the concentrations of 15.9 mg Cu/L were prone to fall out of solution and thus was considered the maximum exposure concentration. These concentrations were derived by diluting 8,000 mg Cu/L of NPs stock solution in water to the appropriate concentrations in the nematode growth media. The concentration of total Cu ions within the CuO NPs stock solution was determined using ICP-MS.

A high-throughput assay was used to assay a large number of offspring for average body length and brood size traits. Testing of animals occurred in microtiter plates (96 wells) using the Complex Object Parametric Analyzer and Sorter (COPAS) BIOSORT (Union Biometrica) as per the previous protocol described in Andersen *et al*. [23]. To begin the experiment, three fourth larval stage hermaphroditic nematodes were sorted into each well containing 50 μL K medium along with enough bacterial lysate for 96 hours [23]. HB101 bacterial lysate was made in large batch form and preserved to reduce assay-to-assay variability. Copper salts and nanoparticles were added to media within plates. All plates were incubated in airtight chambers with damp paper towels at 20°C with shaking at 185 rpm for 96 hours to mix animals and food to prevent hypoxia and starvation [23].

The average number of animals measured for each assay for N2, CB4856, DL238, and JU258 was 567±70.5, 840±91.0, 846±115, and 405±47.6, respectively. Feeding behavior was assayed as pharyngeal pumping based on red fluorescence signal within each nematode after red fluorescent beads (Polysciences) were added to the food source. Brood size was counted as the number of offspring generated by a nematode within the 96-hour experiment. Average body size was measured as ‘time of flight’ as the animals passed through the flow cell. These objects are assumed to be nematode progeny with 99.97% accuracy using the support vector machine described previously [23] and were normalized to number of adults initially transferred to each well of the 96-well plate. Animals were killed with 50 mM sodium azide immediately before introduction into the COPAS BIOSORT. This step ensures that animals were straight prior to length measurements. The COPASutils R package was used to process the data [41].

### Neuron degeneration and stress induction scoring

The transgenic reporter strains BY250, RJ907, and RJ938 contain a green fluorescent protein (GFP) that is expressed under control of the *dat-1* promoter within dopaminergic neurons (See *Caenorhabditis elegans* strains for specifics among the strains). Additionally, the transgenic strain KC136 contains a GFP reporter, in which expression is driven by an *hsp-16*.*2* promoter. Transgenic nematode populations were synchronized using standard alkaline hypochlorite method [42] and allowed to enter L1 stage during overnight incubation without food. L1 nematodes were suspended in 1 mL K medium (supplemented with 5 mg/mL cholesterol and HB101 bacterial lysate prepared as in Andersen *et al*. [23]) containing the respective copper treatment at the desired concentration and were incubated at 20°C on a rotational shaker at 250 rpm for 24 hours. Due to the impermeable nature of the cuticle of *C*. *elegans*, a 24-hour exposure was necessary to observe any effect [43,44]. After copper exposure, animals were gently pelleted at 1,500 rpm for 60 seconds. Supernatant was removed, and nematodes were washed twice with K medium (pH 6.5, with no cholesterol or lysate added) prior to plating onto K medium agar with OP50 bacteria. After a 72-hour recovering period, adult animals were picked onto agar stubs. BY250, RJ907, and RJ938 nematodes were scored positive for neural degeneration if neurons were malformed or completely absent when observed under a fluorescent microscope (Nikon te2000). For stress response, KC136 nematodes were imaged (Leica DMI 6000B inverted microscope, Leica microsystems with Leica Application Suite AF) and GFP expression was measured as voxel volume and normalized to length using Metamorph 5.5 software (Molecular devices). Data represent three independent experiments and at least 40 nematodes were scored for each treatment.

### Statistical Analysis

Statistical analysis was performed in R statistical package [45] using the aov function of ANOVA and TukeyHSD function for follow up Tukey’s honest significant difference test. ANOVA assumptions were examined by Q-Q plot and residual plots in R statistical package. The effects of the treatments on toxicological endpoints (body length, feeding behavior, neural degeneration, etc.) were explored by analysis of variance (ANOVA) using strain, treatment (form of copper as CuO NPs or copper sulfate), and dose (mg Cu/L) as factors (Three-way ANOVA), followed by Tukey HSD to test differences between group means. Toxicological endpoint data were normalized to the data of the untreated nematodes of the respective strain and represented as percent of untreated. Percentage data were subjected to arcsine square root or log transformation prior to analysis. The output of the Complex Object Parametric Analyzer and Sorter (COPAS) BIOSORT (Union Biometrica) is available in supplement files.

## Results

### CuO nanoparticles aggregate and released copper ions

**Fig 1:**
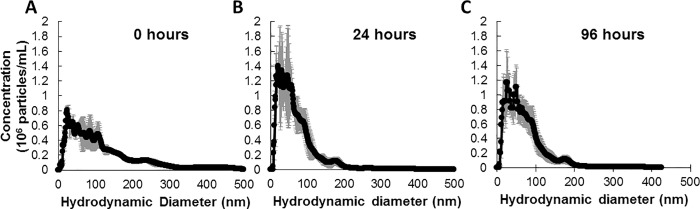
Copper oxide nanoparticles agglomerate in K medium. The hydrodynamic size distribution was observed immediately after suspension of CuO NPs (A), 24 hours after suspension (B), and 96 hours after suspension (C) at 20°C and 250 rpm shaking. Black points and lines represent the mean particle concentration (10^6^ per mL) and the error bars represent the standard error. The copper oxide nanoparticles decreased in average hydrodynamic diameter with time, which indicated a gradual reduction in agglomerate and aggregate size.

**Fig 2:**
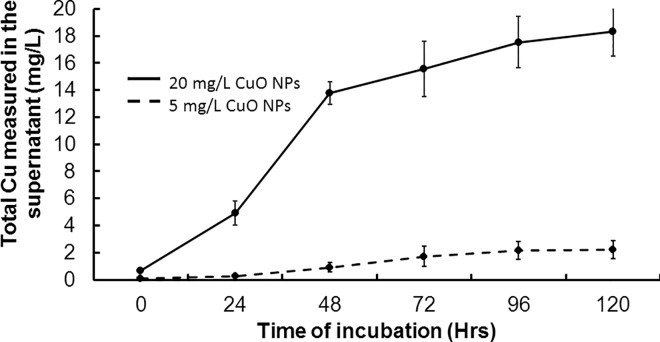
Copper oxide nanoparticles dissolution in K medium (pH 6.5). The concentration of total Cu ions released over time was determined via ICP-MS at different time points. Total Cu concentration (free ions and bound to the media components) begins to plateau at 96 hours of nanoparticle incubation in media. Error bars represent standard error.

The amount of ions released from metal oxide NPs depends on the physicochemical properties (such as size, surface charge, and crystal structure) and is an important factor in the measured toxicity to *C*. *elegans* [5]. The copper oxide (CuO) NPs used throughout this study have been previously characterized by transmission electron microscopy [8] and were observed to be predominantly spherical with a rough surface and an average primary particle diameter of 28.4 nm and a parallel crystal lattice fringe spacing of 2.4 Å. When dispersed in nematode growth K medium, the CuO NPs undergo changes in the hydrodynamic diameter (Fig 1). The increased hydrodynamic diameter is due to CuO NPs interacting with other CuO NPs and salts within the K medium, forming larger and more complex aggregates and agglomerates. CuO NPs could also be interacting with the organic bacterial lysate components, and these agglomerates can subsequently be ingested by the nematodes. After suspending the CuO NPs in K medium, the concentration of the total copper (both free Cu ions and those ions bound to media components) corresponds to 25% of the NPs initial mass after 24-hour incubation and up to 91% of the NPs initial mass after 120-hours (in K medium pH 6.4, Fig 2). No Cu ions were detected when the CuO NPs were suspended in water (pH 6.5, data not shown), suggesting the CuO NPs were not dissolving in water.

### NPs have greater effect on toxicological endpoints compared to copper ions

**Fig 3:**
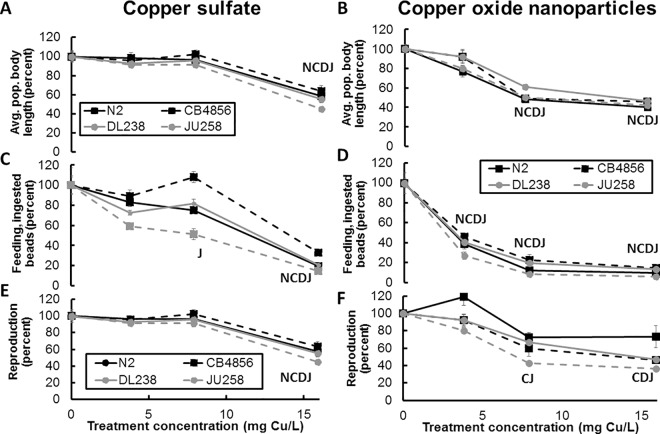
Toxicological endpoint data for the laboratory-adapted *C*. *elegans* N2 strain and wild strains after copper sulfate and copper oxide exposures. The effects on body size (A, B), feeding behavior (C, D), and brood size (E, F) were assayed via the COPAS BIOSORT, and raw data were normalized to values for untreated animals for each respective strain. Data representing endpoint changes after copper sulfate (A, C, E) and copper oxide NPs (B, D, F) exposures. Values significantly different from untreated animals (*p*<0.05) are designated by the first letter of each strain (N for N2 strain, C for CB4856 strain, D for DL238 strain, and J for JU258 strain). Results are presented as mean of four technical replicates and error bars represent standard error.

This study addressed the inhibitory effects of CuO NPs on the physiology of different *C*. *elegans* strains. Several toxicological endpoints were assessed after copper exposures, including average body length as a determinate of developmental stages, the brood size as a quantification of reproduction success, and fluorescent bead ingestion as a measure of feeding behavior of the nematodes (Fig 3, Fig A in S2 File). Values for each toxicological endpoint were normalized to the untreated animals of the same strain, *e*.*g*. CB4856 treated with copper is normalized to untreated CB4856, and represented as a percentage of the untreated traits (Fig 3). The three-way ANOVA revealed that the treatment (form of Cu as CuO NP or copper sulfate), dose of Cu, and strain had a significant, but complex effect on feeding behavior (F_31,92_ = 64.47, p<0.0001), reproduction (F_31,93_ = 16.65, p<0.0001), and body length (F_31,90_ = 32.37, p<0.0001). The greater sensitivity of *C*. *elegans* to CuO NPs exposure compared with copper sulfate highlights the importance of an effect specific to the CuO NPs.

The average body length of a nematode population can be considered a measure of development as stressors can delay development of nematodes at first or second larval stages [46]. These data represent the body lengths of an entire population of animals after 96 hours of incubation, not single animals. Only the first animals laid during the start of the experiment have grown to adults at this time point. The majority of the animals in the experimental treatments are in the early larval stages. Therefore, a nematode population consisting of younger animals would be shorter for average body length. A population consisting primarily of adult nematodes or fourth larval stage animals would be much longer for average body length. The three-way ANOVA showed body length was significantly affected by treatment (form of Cu as CuO NP or copper sulfate, dose of Cu, and strain (p<0.0001). The expected interaction between the dose and treatment was significant (p<0.0001) for body length. The interactions of the treatment and strain (p = 0.068), and the dose of Cu and strain (p = 0.066) were not significantly different for body length. No significant difference was detected in the three-way interaction term of treatment, dose, and strain (p = 0.604) for body length.

All four strains showed a significant difference between treatments (p<0.001, Tukey’s HSD of Strain:Treatment, Table G in S2 File), indicating that a greater decrease in average body length caused by CuO NPs as compared to copper sulfate exists. Untreated *C*. *elegans* N2 populations had an average body length of 226.4 ± 9.5 μm. Treatment with CuO NPs led to a significant decrease (p<0.001 Table A in S2 File) in average body length at the two highest concentrations compared to untreated animals (205.1 and 124.5 μm at 7.9 and 15.9 mg Cu/L, respectively). By contrast, the copper sulfate exposed populations displayed significantly decreased average body length (p<0.001) at only the highest concentrations (216.7 μm at 15.9 mg Cu/L). Significantly shorter body lengths were measured in the *C*. *elegans* N2 populations exposed to CuO NPs compared to animals exposed to copper sulfate (p<0.05 at 7.9 and 15.9 mg Cu/L, Table B in S2 File).

Three wild *C*. *elegans* strains were also exposed and the average body length was analyzed to observe if genetically diverse strains displayed similar trends in sensitivity to CuO NPs. The trend of significantly greater effects (p<0.001, Table B in S2 File) of CuO NPs compared to copper sulfate treatment on the average body length was also observed in the three wild strains at 7.9 mg Cu/L (Fig 3A and 3B) but not significantly different at the highest concentration of 15.9 mg Cu/L (p>0.1, Table B in S2 File). Statistical analysis showed significant differences (p<0.05) in response of body length between the strains with the exception of CB4856 and DL238 (p = 0.334, Tukey’s HSD of Strain; Table D in S2 File). The different effects on body length by treatment are highlighted when directly comparing values from Cu ion exposure to those observed after CuO NPs exposure (Fig B in S2 File). The wild strain average body length decreased when the populations were exposed to moderate concentrations of CuO NPs 7.9 mg Cu/L, but copper sulfate exposure was only significantly inhibitory at the highest tested concentration (p<0.001, 15.9 mg Cu/L).

Feeding behavior has been shown to be of particular importance regarding nematode physiology and behavior [24]. To observe changes in feeding behavior due to copper challenge, red fluorescent beads were introduced into the nematode growth medium (Fig 3C and 3D). As the nematodes feed on bacterial lysate, they also ingest the fluorescent beads; hence the amount of fluorescence was quantified to represent feeding behavior [47]. Bacterial lysate was supplied to the K medium during the exposure assay to ensure the animals did not starve and prevent dauer formation. The three-way ANOVA showed feeding behavior was significantly affected by treatment (form of Cu as CuO NP or copper sulfate, dose of Cu, and strain (p<0.0001). Significant interactions were detected between the form of Cu and strain (p = 0.021), the dose of Cu and strain (p<0.001), and the expected interaction of dose and form of Cu (p<0.0001) for feeding behavior. No significant difference was detected in the three-way interaction term of treatment, dose, and strain (p = 0.784) for feeding behavior.

The N2 strain showed significant (p<0.001, Table A in S2 File) decreases in fluorescent red signal at all exposures of CuO NPs compared to untreated animals (Fig 3D) but was only significantly affected by copper sulfate at the highest concentration (p<0.001, Table A in S2 File, Fig 3C). CuO NPs exposure impacted the feeding behavior of N2 strain significantly more than copper sulfate exposure alone (all concentrations; Table B in S2 File), as measured by a decrease in fluorescence (p<0.05).

A similar trend of increased sensitivity to CuO NPs, as compared with copper sulfate exposure, was observed when assessing feeding behavior in the wild *C*. *elegans* strains. These wild nematode strains displayed significantly greater reduction in red fluorescent signal (p<0.05) after CuO NPs exposure compared to equal molar dose with copper sulfate (7.9 mg Cu/L, Table B in S2 File). Significant difference was detected in feeding behavior between N2 and JU258 compared to any other strain (p<0.05) with similarity between DL238 and CB4856 (p = 0.334, Tukey’s HSD, Strain, Table D in S2 File). The Tukey’s HSD test showed that the interaction between strain and treatment in feeding behavior was significant between treatments in all four strains tested (p<0.001, Table E in S2 File), indicating greater sensitivity to CuO NPs than copper sulfate.

To test if exposure to CuO NPs has inhibitory effects on *C*. *elegans* reproduction, the brood size of all studied strains was quantified after 96 hours of exposure. The average number of progeny in the untreated N2 nematodes was 192±11 (Fig 3E and 3F), similar to results reported by Calafato *et al*. [18]. The three-way ANOVA showed reproduction was significantly affected by treatment (form of Cu as CuO NP or copper sulfate), dose of Cu, and strain (p<0.0001). Significant interactions were detected between the form of Cu and strain (p = 0.002), the dose of Cu and strain (p = 0.029), and expected interaction of dose and form of Cu (p<0.0001) for reproduction. The three-way interaction term was not significant (p = 0.410).

The number of N2 progeny decreased significantly (p<0.001) only after copper sulfate exposure at the highest concentration of copper sulfate (15.9 mg Cu/L). Exposure of the N2 stain to CuO NPs resulted in a variable decrease in brood size that was not statistically different compared to untreated animals (p>0.05; Table A in S2 File). Similar to the N2 strain, the three wild strains also only showed significant decreases in progeny at the highest concentration of copper sulfate (15.9 mg Cu/L, p<0.015; Table C in S2 File). Reproduction was significantly different between the JU258 strain compared to all other strains (p<0.003, Tukey’s HSD, Strain; Table I in S2 File). CB4885, DL238, and N2 were not significantly different in reproduction (p>0.072). Unlike N2 all three wild strains displayed inhibited reproduction after exposure to 7.9 and 15.9 mg Cu/L of CuO NPs (p<0.001). Statistical analyses of the strain and treatment interaction showed significant difference between the CB4856 and JU258 strains (p<0.002) and no significant difference between the N2 and DL238 strains (p>0.308, Tukey’s HSD; Table H in S2 File). This result suggests similar sensitivity among the N2 and wild strains to copper sulfate exposure but a difference in the N2 sensitivity to CuO NPs, as N2 reproduction was never reduced from CuO NPs exposure.

These measurements of selected toxicological endpoints for all studied strains collectively indicated a greater sensitivity to CuO NPs compared to copper sulfate despite the genetic and phenotypic differences between *C*. *elegans* N2 and the wild strains. The observed differences in the degree of sensitivity of the wild strains to copper exposure, compared to N2 at some toxicological endpoints, requires a more in-depth investigation in order to determine the genetic causes.

### CuO nanoparticles affect nematode neuronal morphology

The effect of copper exposure on neuronal health was examined using several transgenic nematode strains. The neuron morphology of a transgenic *C*. *elegans* strain with dopaminergic neurons expressing GFP [32] was assayed after exposure to both CuO NPs and copper sulfate. In addition, two mutant strains containing knockouts of either *smf-1* or *smf-2* were used to examine whether these metal transporters are involved in Cu-induced neurodegeneration after exposure to CuO NPs and copper sulfate.

**Fig 4:**
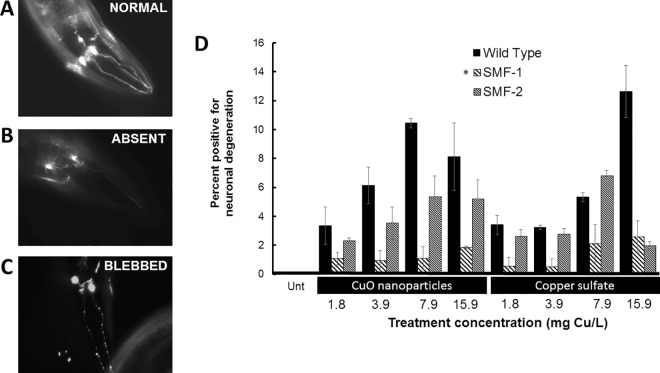
Copper oxide nanoparticles induce an equal degree of dopaminergic (DA) neuron degeneration in *C*. *elegans* BY250 wild-type strain compared to copper sulfate exposure. Fluorescent images depicting healthy DA neurons (A) compared to deformed neurons that were either never formed (B) or degenerated (‘beaded’ or ‘blebbed’) (C). The bar graph represents percent of the nematode population with neuron degeneration, which increases from both copper oxide and copper sulfate treatments (D). “Unt” represents untreated *C*. *elegans* that showed no observable neuron degeneration (n = 203). Results are presented as the mean of three independent experiments with a minimum of 40 nematodes observed per experiment. Two-way ANOVA statistical analysis indicates a significant difference between exposed BY250 population and exposed *smf-1* population, as represented by an asterisk (*). Error bars represent standard error.

Neurodegeneration was observed after copper treatment resulting in alterations in normal neuronal morphology (depicted in Fig 4) in the form of absent neurons or degenerated neurons (“blebbed”) (Fig 4B and 4C). Overall, we found a significant effect of on neural degeneration (three-way ANOVA, F_23,48_ = 11.23, p<0.0001) with strain and dose of Cu having the highest impact (p<0.0001) and form of Cu was not significant (p = 0.410). A significant interaction between dose and strain (p = 0.002) was detected. The neurodegeneration observed in treated animals occurred in a dose-dependent manner in 3–12% of the population examined. Exposure to CuO NPs resulted in a greater amount of neurodegeneration (in 6.4% and 10.4% of the scored animals; n = 203 and 181, respectively) compared with copper sulfate (in 3.2% and 5.3% of the scored animals; n = 213 and 266, respectively) at two treatments (3.8 and 7.9 mg Cu/L, Fig 4) with equal copper content. After copper exposure, the *smf-1* strain, which contains a deletion of a divalent metal transporter gene, displayed neurodegeneration in a significantly smaller percent of the population compared to BY250 wild-type strain (p<0.001, TukeyHSD of Strain; Table J in S2 File). Neuron degeneration was absent and not detected in the untreated animals for any transgenic strain (Untreated, Fig 4).

### CuO nanoparticles induce stress responses

**Fig 5:**
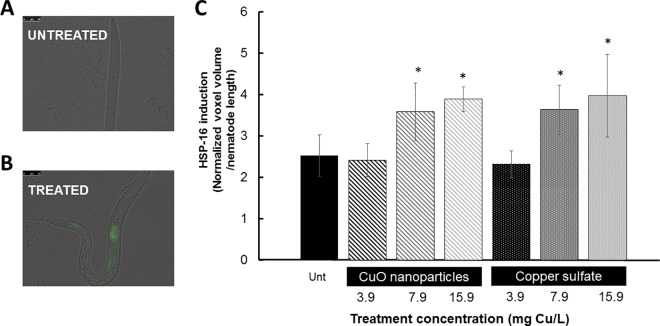
Significant stress induction, as measured by *hsp-16*.*2* induction of GFP in transgenic *C*. *elegans* strains, was observed after both copper oxide nanoparticle and copper sulfate exposures. Fluorescent images depict endogenously regulated GFP expression (A) and an increase in the induction of GFP (B). The bar graph is representative of experiments with at least 25 nematodes examined per treatment. The graph depicts increased GFP expression after both copper oxide nanoparticles and copper sulfate at the two highest concentrations employed (C). Significant results as compared to untreated (Tukey’s HSD *p*<0.05) are marked with asterisks (*). Error bars represent standard error.

The response of *C*. *elegans* to CuO NPs and copper sulfate exposures was assayed using a reporter strain with GFP expression driven by a heat-shock inducible stress promoter (*hsp-16*.*2)*. Exposure to both CuO NPs and copper sulfate resulted in the induction of stress response in *C*. *elegans*, as indicated by the increased GFP expression of the *hsp-16*.*2* reporter strain in comparison to untreated nematodes (Fig 5). Increased GFP expression may be considered organismal stress response from protein damage and unfolding because HSP-16.2 is a small heat-shock induced chaperone and is considered a general stress indicator reacting to several stressors, including temperature and oxidative stresses [40].

## Discussion

### Copper ions might interact with the food source

Because the CuO NPs suspensions contained significantly fewer copper ions compared to the copper sulfate exposure (*p<*0.04) but caused equal or greater effect at several toxicological endpoints, these data support the greater sensitivity of *C*. *elegans* to CuO NPs exposure compared with copper sulfate. The concentration of copper sulfate employed in this study was approximately two-fold greater than the Cu ion concentration released from the CuO NPs after 24-hour incubation in K medium (Fig 2). It is important not to underestimate the impact of the bacterial cells on the chemistry occurring with Cu ion complexation. If live bacteria were used as a food source, free Cu ions in solution could also have been taken up by the bacteria. Our experiments were performed with lysed bacterial cells in an attempt to remove the interaction of Cu ions with bacterial cells. However, studies of surface acid-base properties of bacterial cells walls suggest the carboxyl, hydroxyl, phosphate, amino, and amide functional groups on the surface of bacteria display biosorptive behavior for Cu(II) and Pb(II) ions [48]. An increase in amine and carboxylate groups at the *Penicillium chrysogenum* by cell surface modification also resulted in a significant increase in sorption capacity for Cu(II), Pb(II), and Ni(II) ions [49]. The evidence of interactions between metal ions and bacterial cellular proteins indicate that the doses of exposure used within this study may be overestimated.

### CuO nanoparticles strongly inhibit food intake

Food ingestion and, therefore, energy uptake are essential for every animal and impairment can decrease survival and fitness. A reduction in nematode feeding behavior has been suggested to be both a response to environmental stress through neurotransmitters and changes in pharyngeal activity [50]. Limiting food uptake has been linked to increased sensitivity to stress and a reduction in motility in *C*. *elegans* [51]. The feeding behavior was the most sensitive toxicological endpoint in our study as the CuO NPs at every tested concentration displayed a significant effect (Table A in S2 File). A trend of increased sensitivity to CuO NPs compared to copper sulfate was observed when assessing feeding behavior in all tested *C*. *elegans* strains (Fig 3, Table B in S2 File). Food-borne exposure of copper chloride has been shown to be the primary, and most toxic, route of exposure in *C*. *elegans* [52]. CuO NPs ingestion is most likely the point of entry of the NPs in *C*. *elegans* in our study, because the nematode cuticle is poorly permeable to many chemicals [44].

The introduction of nanoparticles into *C*. *elegans* from accidental ingestion, along with the bacterial food source, might also be possible and has been highlighted in other studies [53]. Silver NPs exposure without bacteria in the growth medium resulted in significantly lower toxicity compared to NPs exposure along with the addition of food. The ingestion of NPs is a frequent route of exposure in *Daphnia magna*. After exposure to CuO or TiO_2_ NPs, it was observed that NPs were accumulating within the midgut of *Daphnia* [54]. Once ingested, pH changes and enzymatic activity from the stomach/midgut could result in increased NPs dissolution and ion release [55]. An effect on *C*. *elegans* feeding behavior has also been observed after exposure to other toxicants, including silver NPs [56], methyl mercury [57], salicylate, high heat, and sulfhydryl-reactive compounds [56]. This reduction of feeding may in part be a defense mechanism of the animal in order to reduce toxicant intake as this reduction in pharyngeal pumping has been observed after treatment with heavy metals [52].

### CuO NPs cause nematode development delay

Greater sensitivity to CuO NPs exposure compared to exposure with copper sulfate was observed in all three wild and N2 strains based on reduction in the average body length as a measure of developmental stage. The N2 *C*. *elegans* strain displayed a more sensitive response to CuO NPs exposure because a significant reduction in body size was observed with 3.9 mg Cu/L exposure but was not observed in the wild strains until 7.9 mg Cu/L. The observed differences to copper exposure in the N2 compared to the wild strains may represent the impact of laboratory adaptation on the N2 strain. The developmental delay of *C*. *elegans* is a common physiological response to stress and has been observed after exposure to copper sulfate [44], titanium dioxide (TiO_2_), and zinc oxide (ZnO) NPs [58]. Wu *et al*. observed the ZnO NPs to be more toxic than TiO_2_, which could be caused by greater metal ion release measured in the ZnO NPs suspensions compared to TiO_2_ NPs [58].

Previous studies have shown that *C*. *elegans* displayed similar phenotypes, *e*.*g*. both growth delay and reduced lifespan, after exposure to copper ions from copper salt [18]. Changes in the body length of individual nematodes have been linked to decreased food intake, as well as effects from perturbation of insulin IGF-1 signaling [59]. Therefore, a portion of the reduced average body length observed in this study might be a consequence from the smaller individual body size as a result of diminished feeding. Additionally, a portion of the nematode population could be entering the dauer stage after encountering the copper stress, further complicating the interpretation of these data. Thus, multiple physiological effects triggered by the CuO NPs and copper ion exposures might be contributing to the observed *C*. *elegans* developmental delay.

### Nematode reproduction is affected after copper exposure

Reproduction is a critical endpoint to analyze because it has been shown to be sensitive to lower concentrations of chemical stressors compared to concentrations that affect *C*. *elegans* behavior and viability [10]. However, within our study, the reproduction of the wild strains was only reduced significantly after 96 hours of exposure to CuO NPs and the reproduction of all strains after exposure to copper sulfate at the highest concentrations (15.9 mg Cu/L, Fig 3E and 3F) used in this study. The cause of the reduced nematode brood size may be caused by either a decreased number of adults capable of laying eggs or an increased amount of sterility in the adult worms. A decrease in the reproductive capabilities of *C*. *elegans*, in the form of decreased rate of egg laying or decreased embryo survival, has been observed after exposure to fullerene NPs [60] and silver NPs [61]. Elevated levels of copper ions have been observed to induce paralysis [51], which may reduce feeding and stress the animal to the point of affecting reproduction.

### Wild nematode strains have differential responses to CuO NPs and copper sulfate when compared to the laboratory-adapted strain

The use of wild strains genetically diverse from a laboratory-adapted strain enabled a more complete assessment of the toxicological effects of CuO nanoparticles on *C*. *elegans*. Increased sensitivity to CuO NPs exposure compared to copper sulfate was observed in almost all *C*. *elegans* strains and concentrations examined based on the toxicological endpoints of reproduction, feeding behavior, and average body length (Fig 3, Table B in S2 File). When comparing the response of the wild strains to that of the laboratory-adapted strain, the N2 strain was more resistant to copper stress based on the N2 strain response to feeding and reproduction. For example, the brood size of N2 was not significantly affected by CuO NPs exposure, but the wild strains displayed significant reductions at the highest concentration (15.9 mg Cu/L, Table C in S2 File). The feeding of JU258 was impaired by all concentrations of copper sulfate, but N2 was only affected by the highest copper sulfate exposure. Conversely, the feeding of CB4856 was impaired less than all the other strains at all concentrations of copper sulfate.

At the same time, significant reduction in body size was measured at 3.9 mg Cu/L in the N2 strain that was not observed in the wild strains until 7.9 mg Cu/L. These differential results between the laboratory-adapted strain and the wild strains highlight the importance of strain variation in toxicological studies as previously shown [23,27,38] but also highlight the necessity to measure more toxicological endpoints at a wide range of exposure concentrations.

The *C*. *elegans* N2 strain has been used in the laboratory for decades and has accumulated several phenotypic and genetic differences when compared to recently isolated wild strains [24,62][21]. Some known phenotypic differences in the N2 strain compared to other *C*. *elegans* strains have been linked to changes in feeding behavior [24]. The differences in physiology and behavior of wild *C*. *elegans* strains compared to the N2 strain can include aggregation behavior observed during feeding [22] as well as copulatory plug formation and defects in the tail structure significant enough to reduce mating ability in males [63]. The wild strain CB4856 has a high level of genetic divergence from N2 for a *C*. *elegans* strain, averaging a SNP every 835 bp compared to N2 [64]. The increased sensitivity of CB4856 feeding behavior could be an example of genetic variants affecting toxicity or could also be random chance or due to an environmental factor, thus supporting the need for future studies with varying strains.

### Copper exposures cause neuron degeneration

The potential of CuO NPs to influence the nematode neuronal morphology was assayed utilizing transgenic strains of *C*. *elegans* with neuron-specific proteins tagged with GFP to visualize dopaminergic neuron degeneration after copper exposure. The association of copper ions with neuronal degeneration has been established in *C*. *elegans* and humans [65,66]. Transgene expression of genes of interest can provide more specific information regarding bioavailability and the phenotype of the NPs effect compared to conventional endpoint assessment [33,39]. The CuO NPs induced morphological changes to neuronal cells in a small portion of the *C*. *elegans* populations in a concentration-dependent manner. The neurodegeneration caused by CuO NPs compared to copper sulfate exposure was not statistically different, suggesting the released Cu ions may be the sole source of neuron damage (Table D in S2 File).

### Location and expression of SMF-1 may play a role in reduced neurodegeneration

Copper treatment of *C*. *elegans* strains containing single-gene knockout mutations in the metal ion transporter *smf-1* displayed reduced neurodegeneration supporting a Cu ion effect. The differential sensitivity between *smf-1* and *smf-2* mutant strains to Cu ions may be the result of differential localization of the two metal transporters or different abundance of these transporters.

The difference in sensitivity observed between *smf-1* and *smf*-2 knockout mutants may be the result of reduced Cu ion transport within the intestine as well as reduced Cu ion intake within neuronal cells. Translational GFP fusions revealed SMF-1 is greatly expressed in several different cell types, including within the gut epithelium and weakly expressed within head neurons [46]. In comparison, SMF-2 was found in only a few cell types, specifically the pharynx and pharyngeal-intestinal valve cells. In addition to the different cell type locations of SMF-1 and SMF-2, the two transporters are also located in different regions within the cells. The SMF-1 transporter was mainly found at the apical plasma membrane while SMF-2 was mainly observed within intracellular cytoplasmic compartments [46]. This difference in localization within the cell might further explain the observed differential sensitivity between *smf-1* and *smf-2* mutant strains to Cu exposure. The exposure to Cu ions from NPs is most likely occurring via highly localized and concentrated release of Cu ions from the surface of the CuO NPs after being ingested by the animal.

Toxicity from copper sulfate exposure has been shown to be mediated by HSP-16.2 [16]. Similarly to the observed effect on neuronal health, the comparable induction of the *hsp-16*.*2* promoter during exposures of CuO NPs and copper sulfate suggests a stress response due to the effect of free Cu ions from the salt or released from CuO NPs.

## Conclusion

This study describes the physiological effects of CuO NPs to *C*. *elegans* N2 and a genetically diverse set of wild nematode strains as observed by inhibitory effects on feeding, reproduction, development, and neuron morphology. The results support an increased sensitivity to CuO NPs compared to copper sulfate in a genetically broad group of *C*. *elegans* strains. CuO NPs sensitivity was a trait observed in all *C*. *elegans* strains assayed despite their different genotypes, suggesting this effect is not due to laboratory domestication of the N2 strain. The nanoparticle-specific effects contributed to the toxicity observed in *C*. *elegans* growth, reproduction, and feeding possible from nanoparticles affecting only the gut-proximal cells. Though some effects on *C*. *elegans* were attributed to be nanoparticle-specific, other assays suggest an effect only from the released Cu ions. Neuronal deformation occurred in a similar portion of the *C*. *elegans* population after exposure with either CuO NPs or copper sulfate at equal amounts of copper. Similarly, an equal response of *hsp-16*.*2* expression to copper stress was observed in the *C*. *elegans* population after CuO NPs and soluble copper exposures. We believe that the released copper ions from CuO NPs are a major factor contributing to the observed NPs effect on neuronal health and organismal stress response.

## Supporting Information

S1 File Raw data (XLSX)

S2 File Supplemental figures and tables: Figure A: Filtration could remove Cu ions from supernatant. Figure B: Comparison of copper exposures, copper sulfate and copper oxide, on N2 and three wild *Caenorhabditis elegans* strains. Table A. Statistical difference between untreated and copper exposed nematodes. Table B. Statistical differences between copper oxide nanoparticle inhibitory effects and the inhibitory effect from copper sulfate exposure. Table C. Statistical differences in response to copper exposure from the lab adapted N2 strain and the wild nematode strains. Table D. Tukey’s HSD statistical analysis results post 3-WAY ANOVA based on the main factor of strain for feeding behavior. Table E. Tukey’s HSD statistical analysis results post 3-WAY ANOVA based on the interaction of strain and treatment (form of Cu) for feeding behavior. Table F. Tukey’s HSD statistical analysis results post 3-WAY ANOVA based on the main factor of strain for average body length. Table G. Tukey’s HSD statistical analysis results post 3-WAY ANOVA based on the interaction of strain and treatment (form of Cu) for average body length. Table H. Tukey’s HSD statistical analysis results post 3-WAY ANOVA based on the main factor of strain for reproduction. Table I. Tukey’s HSD statistical analysis results post 3-WAY ANOVA based on the interaction of strain and treatment (form of Cu) for reproduction. Table J. Tukey’s HSD statistical analysis results post 3-WAY ANOVA based on the main factor of strain for neuron degeneration. Table K. Tukey’s HSD statistical analysis results post 3-WAY ANOVA based on the interaction of strain and treatment (form of Cu) for neuron degeneration.
(PDF)
