# Supplementary material for: Copper Oxide Nanoparticles Impact Several Toxicological Endpoints and Cause Neurodegeneration in Caenorhabditis elegans
Source: PLoS One. 2016 Dec 2;11(12):e0167613. doi: 10.1371/journal.pone.0167613 (PMC5135131; doi:10.1371/journal.pone.0167613)
Supplement: S2 File — Supplemental figures and tables: Figure A: Filtration could remove Cu ions from supernatant. Figure B: Comparison of copper exposures, copper sulfate and copper oxide, on N2 and three wild Caenorhabditis elegans strains. Table A. Statistical difference between untreated and copper exposed nematodes. Table B. Statistical differences between copper oxide nanoparticle inhibitory effects and the inhibitory effect from copper sulfate exposure. Table C. Statistical differences in response to copper exposure from the lab adapted N2 strain and the wild nematode strains. Table D. Tukey’s HSD statistical analysis results post 3-WAY ANOVA based on the main factor of strain for feeding behavior. Table E. Tukey’s HSD statistical analysis results post 3-WAY ANOVA based on the interaction of strain and treatment (form of Cu) for feeding behavior. Table F. Tukey’s HSD statistical analysis results post 3-WAY ANOVA based on the main factor of strain for average body length. Table G. Tukey’s HSD statistical analysis results post 3-WAY ANOVA based on the interaction of strain and treatment (form of Cu) for average body length. Table H. Tukey’s HSD statistical analysis results post 3-WAY ANOVA based on the main factor of strain for reproduction. Table I. Tukey’s HSD statistical analysis results post 3-WAY ANOVA based on the interaction of strain and treatment (form of Cu) for reproduction. Table J. Tukey’s HSD statistical analysis results post 3-WAY ANOVA based on the main factor of strain for neuron degeneration. Table K. Tukey’s HSD statistical analysis results post 3-WAY ANOVA based on the interaction of strain and treatment (form of Cu) for neuron degeneration. (PDF) [file pone.0167613.s002.pdf]

Supplemental Information

Copper oxide nanoparticles impact several toxicological endpoints and cause neurodegeneration in *Caenorhabditis elegans*

Michael J. Mashock[1], Tyler Zanon[1], Anthony D. Kappell[1], Lisa N. Petrella[1], Erik C. Andersen[2], and Krassimira R. Hristova[1*]

[1]Department of Biological Sciences, Marquette University, Milwaukee, Wisconsin, United States of America

[2]Department of Molecular Biosciences, Northwestern University, Evanston, Illinois, United States of America

[*] Corresponding author

Email: krassimira.hristova@marquette.edu

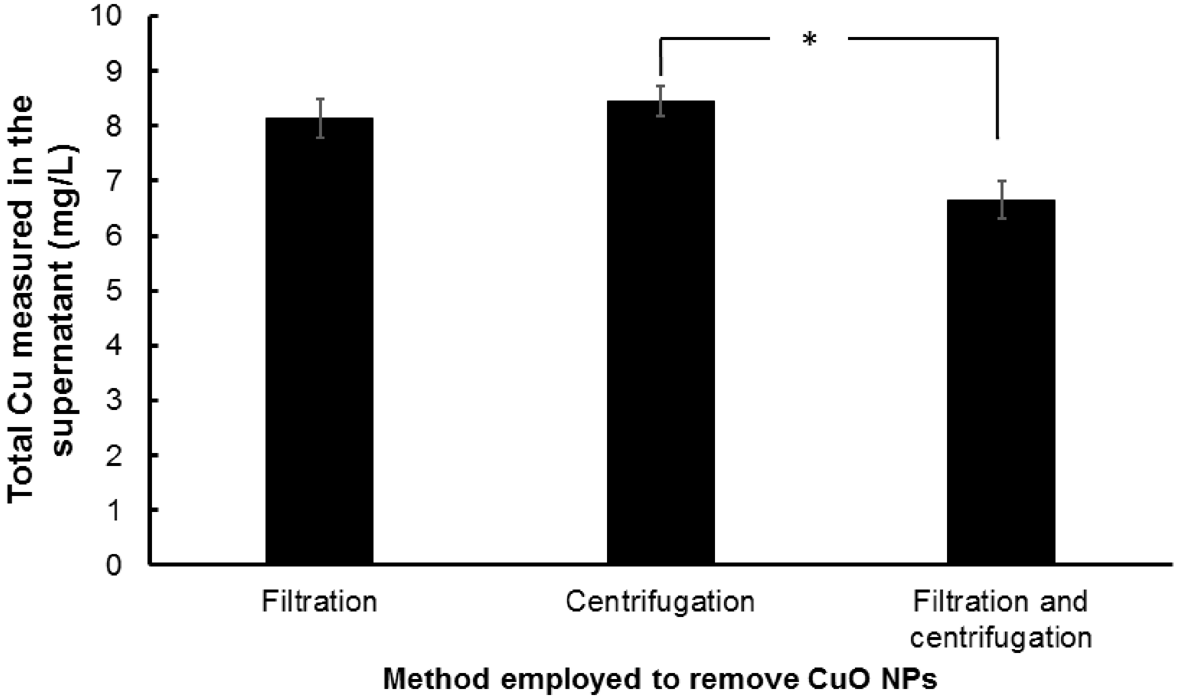

Figure A: Filtration could remove Cu ions from supernatant. The total amount of Cu ions released over time was determined via ICP-MS after 24-hour incubation of CuO NPs (9 mg Cu/L) in K-media. Statistical analysis (two-way ANOVA) indicates a significant difference between supernatant that underwent both filtration as well as centrifugation and supernatant that only underwent centrifugation, as represented by an asterisk (*). Error bars represent standard error.

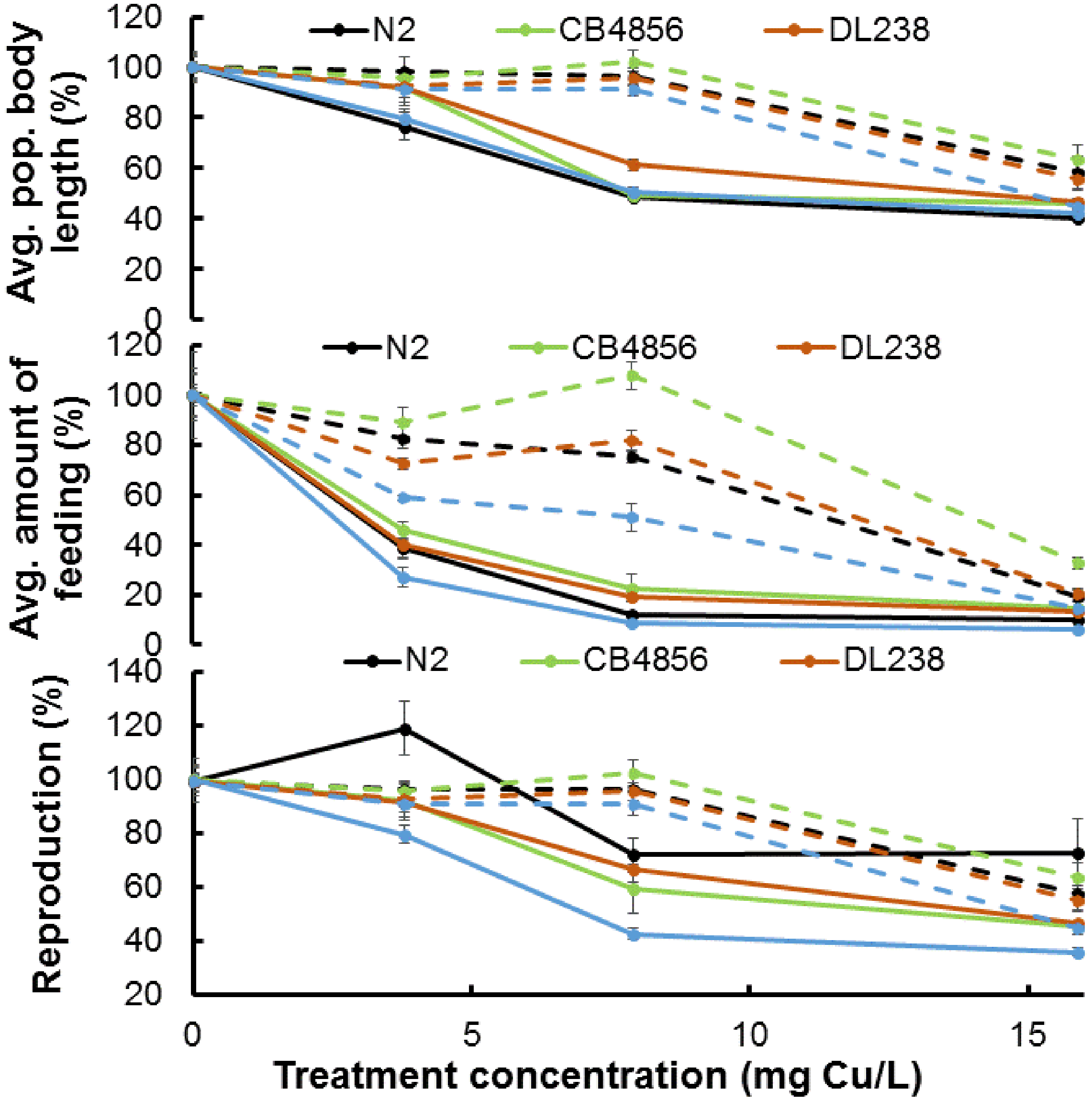

Figure B: Comparison of copper exposures, copper sulfate and copper oxide, on N2 and three wild *Caenorhabditis elegans* strains. The effects on body size (A), feeding behavior (B), and brood size (C) were examined. Data from endpoint changes are represented by dashed lines for copper sulfate exposure and solid lines for copper oxide NPs exposure. Results are presented as mean of four technical replicates and error bars represent standard error.

Strain	mg Cu/L	Toxicological Endpoint Measurment					
		Reproduction		Feeding Behavior		Average Body Length	
		NP	Cu	NP	Cu	NP	Cu
N2	3.8	1.000	1.000	<0.001	1.000	0.247	1.000
	7.9	0.365	1.000	<0.001	0.997	<0.001	1.000
	15.9	0.190	<0.001	<0.001	<0.001	<0.001	<0.001
CB4856	3.8	1.000	1.000	<0.001	1.000	1.000	1.000
	7.9	<0.001	1.000	<0.001	1.000	<0.001	1.000
	15.9	<0.001	0.015	<0.001	<0.001	<0.001	<0.001
DL238	3.8	1.000	1.000	<0.001	0.998	1.000	1.000
	7.9	0.075	1.000	<0.001	1.000	<0.001	1.000
	15.9	<0.001	<0.001	<0.001	<0.001	<0.001	<0.001
JU258	3.8	0.970	1.000	<0.001	0.127	0.669	1.000
	7.9	<0.001	1.000	<0.001	0.004	<0.001	1.000
	15.9	<0.001	<0.001	<0.001	<0.001	<0.001	<0.001

Table A. Statistical difference between untreated and copper exposed nematodes. Statistical comparison, Tukey's honest significant difference (HSD) p-values, of 24-hour copper exposed *Caenorhabditis elegans* and untreated nematode population body length, feeding behavior, and reproduction. The p-values were determined by Tukey's HSD using R; $p<0.05$ was considered significant.

Strain	mg Cu/L	Toxicological Endpoint Measurment		
		Reproduction	Feeding Behavior	Average Body Length
N2	3.8	0.993	<0.001	0.380
	7.9	0.586	<0.001	<0.001
	15.9	0.995	0.014	0.026
CB4856	3.8	1.000	0.004	1.000
	7.9	<0.001	<0.001	<0.001
	15.9	0.591	<0.001	0.107
DL238	3.8	1.000	0.155	1.000
	7.9	0.202	<0.001	0.002
	15.9	1.000	0.980	0.997
JU258	3.8	1.000	0.080	0.999
	7.9	<0.001	<0.001	<0.001
	15.9	0.978	0.035	0.720

Table B. Statistical differences between copper oxide nanoparticle inhibitory effects and the inhibitory effect from copper sulfate exposure.

Statistical comparison, Tukey's HSD p-values, of copper oxide nanoparticle and copper sulfate effects after 24-hour exposure on *Caenorhabditis elegans* average body length, feeding behavior, and reproduction. The p-values were determined by Tukey's HSD using R; $p<0.05$ was considered significant.

Strain	Toxicological Endpoint Measurment	Treatment					
		NP (mg Cu/L)			Cu (mg Cu/L)		
		3.8	7.9	15.9	3.8	7.9	15.9
CB4856	Body Length	0.967	1.000	1.000	1.000	1.000	1.000
	Feed Behav.	1.000	0.543	0.981	1.000	0.850	0.124
	Reproduction	0.955	0.985	0.094	1.000	1.000	1.000
DL238	Body Length	0.936	0.873	0.998	1.000	1.000	1.000
	Feed Behav.	1.000	0.080	0.561	1.000	1.000	1.000
	Reproduction	0.889	1.000	0.281	1.000	1.000	1.000
JU258	Body Length	1.000	1.000	1.000	1.000	1.000	0.513
	Feed Behav.	1.000	1.000	1.000	0.915	0.634	0.996
	Reproduction	0.125	0.013	<0.001	1.000	1.000	0.954

Table C. Statistical differences in response to copper exposure from the lab adapted N2 strain and the wild nematode strains.

Statistical comparison, Tukey's HSD p-values, of the laboratory-adapted *Caenorhabditis elegans* N2 strain and the wild nematode strain average body length, feeding behavior, and reproduction after copper exposure. The laboratory-adapted N2 (Bristol) strain and three wild strains were exposed to copper oxide nanoparticles or soluble copper ($CuSO_4$). The p-values were determined by Tukey's HSD using R; $p<0.05$ was considered significant.

Strain	CB4855	JU258	DL238
N2	<0.001	0.038	0.046
CB4856		<0.001	0.334
JU258			<0.001

Table D. Tukey's HSD statistical analysis results post 3-WAY ANOVA based on the main factor of strain for feeding behavior.

Statistical comparison, Tukey's HSD p-values, of the laboratory-adapted *Caenorhabditis elegans* N2 strain and the wild nematode strain feeding behavior. The laboratory-adapted N2 (Bristol) strain and three wild strains were exposed to copper oxide nanoparticles or soluble copper ($CuSO_4$). The p-values were determined by Tukey's HSD using R; $p<0.05$ was considered significant.

Strain		N2	CB4855		DL238		JU258	
	Metal	Cu	NP	Cu	NP	Cu	NP	Cu
N2	NP	<0.001	0.075	<0.001	0.009	<0.001	0.996	<0.001
	Cu		<0.001	0.052	<0.001	1.000	<0.001	0.043
CB4855	NP			<0.001	0.998	<0.001	0.011	<0.001
	Cu				<0.001	0.534	<0.001	<0.001
DL238	NP					<0.001	<0.001	0.005
	Cu						<0.001	0.042
JU258	NP							<0.001

Table E. Tukey's HSD statistical analysis results post 3-WAY ANOVA based on the interaction of strain and treatment (form of Cu) for feeding behavior.

Statistical comparison, Tukey's HSD p-values, of the laboratory-adapted *Caenorhabditis elegans* N2 strain and the wild nematode strain feeding behavior. The laboratory-adapted N2 (Bristol) strain and three wild strains were exposed to copper oxide nanoparticles or soluble copper ($CuSO_4$). The p-values were determined by Tukey's HSD using R; $p<0.05$ was considered significant.

Strain	CB4855	JU258	DL238
N2	0.380	0.105	0.143
CB4856		0.001	0.947
JU258			<0.001

Table F. Tukey's HSD statistical analysis results post 3-WAY ANOVA based on the main factor of strain for average body length.

Statistical comparison, Tukey's HSD p-values, of the laboratory-adapted *Caenorhabditis elegans* N2 strain and the wild nematode strain for average body length. The laboratory-adapted N2 (Bristol) strain and three wild strains were exposed to copper oxide nanoparticles or soluble copper ($CuSO_4$). The p-values were determined by Tukey's HSD using R; $p<0.05$ was considered significant.

Strain		N2	CB4855		DL238		JU258	
	Metal	Cu	NP	Cu	NP	Cu	NP	Cu
N2	NP	<0.001	0.711	<0.001	0.022	<0.001	0.963	<0.001
	Cu		<0.001	0.999	0.021	1.000	<0.001	0.374
CB4855	NP			<0.001	0.691	<0.001	0.135	0.056
	Cu				0.003	0.990	<0.001	0.112
DL238	NP					0.039	<0.001	0.892
	Cu						<0.001	0.522
JU258	NP							<0.001

Table G. Tukey's HSD statistical analysis results post 3-WAY ANOVA based on the interaction of strain and treatment (form of Cu) for average body length.

Statistical comparison, Tukey's HSD p-values, of the laboratory-adapted *Caenorhabditis elegans* N2 strain and the wild nematode strain for average body length. The laboratory-adapted N2 (Bristol) strain and three wild strains were exposed to copper oxide nanoparticles or soluble copper ($CuSO_4$). The p-values were determined by Tukey's HSD using R; $p<0.05$ was considered significant.

Strain	CB4855	JU258	DL238
N2	0.072	<0.001	0.151
CB4856		0.009	0.987
JU258			0.003

Table H. Tukey's HSD statistical analysis results post 3-WAY ANOVA based on the main factor of strain for reproduction.

Statistical comparison, Tukey's HSD p-values, of the laboratory-adapted *Caenorhabditis elegans* N2 strain and the wild nematode strain for reproduction. The laboratory-adapted N2 (Bristol) strain and three wild strains were exposed to copper oxide nanoparticles or soluble copper ($CuSO_4$). The p-values were determined by Tukey's HSD using R; $p<0.05$ was considered significant.

Strain		N2	CB4855		DL238		JU258	
	Metal	Cu	NP	Cu	NP	Cu	NP	Cu
N2	NP	0.999	0.002	1.000	0.081	0.999	<0.001	0.440
	Cu		0.013	0.999	0.268	1.000	<0.001	0.794
CB4855	NP			0.002	0.940	0.017	0.220	0.451
	Cu				0.073	0.998	<0.001	0.413
DL238	NP					0.308	0.010	0.988
	Cu						<0.001	0.830
JU258	NP							<0.001

Table I. Tukey's HSD statistical analysis results post 3-WAY ANOVA based on the interaction of strain and treatment (form of Cu) for reproduction.

Statistical comparison, Tukey's HSD p-values, of the laboratory-adapted *Caenorhabditis elegans* N2 strain and the wild nematode strain for reproduction. The laboratory-adapted N2 (Bristol) strain and three wild strains were exposed to copper oxide nanoparticles or soluble copper ($CuSO_4$). The p-values were determined by Tukey's HSD using R; $p<0.05$ was considered significant.

Strain	RJ907 (SMF1)	RJ938 (SMF2)
BY250	<0.001	0.399
RJ907 (SMF1)		<0.001

Table J. Tukey's HSD statistical analysis results post 3-WAY ANOVA based on the main factor of strain for neuron degeneration.

Statistical comparison, Tukey's HSD p-values, of the laboratory-adapted *Caenorhabditis elegans* N2 strain and the wild nematode strain for neuron degeneration. The laboratory-adapted N2 (Bristol) strain and three wild strains were exposed to copper oxide nanoparticles or soluble copper ($CuSO_4$). The p-values were determined by Tukey's HSD using R; $p<0.05$ was considered significant.

Strain		BY250	RJ907 (SMF1)		RJ938 (SMF2)	
	Metal	Cu	NP	Cu	NP	Cu
BY250	NP	0.460	<0.001	<0.001	0.203	0.760
	Cu		0.002	<0.001	0.996	0.997
SMF1	NP			0.990	0.008	<0.001
RJ907 (SMF1)	Cu				0.001	<0.001
RJ938 (SMF2	NP					0.920

Table K. Tukey's HSD statistical analysis results post 3-WAY ANOVA based on the interaction of strain and treatment (form of Cu) for neuron degeneration.

Statistical comparison, Tukey's HSD p-values, of the laboratory-adapted *Caenorhabditis elegans* N2 strain and the wild nematode strain for neuron degeneration. The laboratory-adapted N2 (Bristol) strain and three wild strains were exposed to copper oxide nanoparticles or soluble copper ($CuSO_4$). The p-values were determined by Tukey's HSD using R; $p<0.05$ was considered significant.
